# Intensive Care Unit Nurses’ Views on Recognizing and Caring for Inpatients’ Anxiety Levels: A Qualitative Descriptive Study

**DOI:** 10.3390/healthcare14193301

**Published:** 2026-10-03

**Authors:** Yüksel Can Öz, Tuba Yilmaz Bulut, Esin Tekin

**Affiliations:** 1 Department of Psychiatric, Faculty of Health Sciences, Kocaeli University, 41380 Kocaeli, Türkiye; yukselcan@kocaeli.edu.tr; 2 Department of Public Health, Faculty of Health Sciences, Kocaeli University, 41380 Kocaeli, Türkiye; 3 Department of Psychiatric, Balıkesir Burhaniye State Hospital, 10700, Balıkesir, Türkiye; esin.tekin.52@gmail.com

**Keywords:** intensive care, anxiety, nursing care, qualitative research

## Abstract

**Highlights:**

**What are the main findings?**

- Intensive care nurses primarily prioritize patients’ urgent physical and clinical needs, which may result in anxiety and other psychological needs receiving less attention.
- Nurses recognize patient anxiety through observation, communication, and patients’ responses to care and treatment.
- Providing information, therapeutic communication, and emotional support were identified as key nursing approaches to reducing patient anxiety.
- Workload, organizational and work-related factors, and insufficient training were identified as important barriers to the assessment and management of anxiety in intensive care settings.

**What are the implications of the main findings?**

- Psychosocial care and systematic anxiety assessment should be more strongly integrated into routine nursing care in intensive care units.
- Intensive care nurses may benefit from in-service training focused on recognizing anxiety, therapeutic communication, emotional support, and anxiety management.
- Organizational strategies addressing workload and work-related barriers may facilitate nurses’ ability to assess and respond to patients’ psychological needs.
- Undergraduate and postgraduate nursing education should place greater emphasis on psychosocial care and the assessment and management of anxiety in critically ill patients.

**Abstract:**

**Background/Objectives**: Anxiety in intensive care patients is a significant concern, yet limited evidence exists regarding nurses’ perspectives on its assessment and management. This study aimed to explore intensive care nurses’ views and experiences regarding the recognition, assessment, and management of anxiety among hospitalized intensive care patients. **Methods**: A qualitative descriptive design was employed, and the study sample consisted of 21 nurses selected from 50 nurses working in the intensive care unit of the hospital. The data were collected through in-depth interviews conducted in person between July and September 2021. The data were analyzed manually using an inductive thematic analysis approach. **Results**: A thorough analysis of the interviews yielded four overarching themes: the nurses’ initial assessment priorities, the nurses’ recognition of and responses to patient anxiety, approaches to reducing anxiety, and factors affecting anxiety assessment. Four main themes and ten sub-themes emerged from the interviews. The theme “Nurses’ Initial Assessment Priorities” comprised the sub-themes “Prioritizing the Emergency” and “Getting to Know the Patient.” The theme “Nurses’ Recognition of and Responses to Patient Anxiety” included “Under-recognition of Anxiety,” “Compliance with Care and Treatment,” and “Recognizing Psychological Needs.” The theme “Approaches to Reducing Anxiety” comprised “Providing Information” and “Therapeutic Communication and Emotional Support.” Finally, the theme “Factors Affecting Anxiety Assessment” included “Workload,” “Organizational and Work-Related Factors,” and “Training Needs.” **Conclusions**: It is recommended that in-service training programmes covering psychosocial care, anxiety assessment and management be organised for intensive care nurses and that more emphasis be placed on psychosocial care and patient anxiety in intensive care in nursing undergraduate and graduate education programmes.

## 1. Introduction

It is evident that every illness experienced engenders a crisis-like situation by disrupting the individual’s physical and emotional equilibrium [1]. Irrespective of its aetiology, illness is a multifaceted concept that has a detrimental effect on the individual in biological, emotional, psychological and social terms [2]. It is important to note that humans are biopsychosocial beings, meaning that any health problem experienced in one area can have a negative effect on other areas. Although individuals may maintain a healthy lifestyle, unexpected events such as acute illness, trauma, or critical health conditions can lead to a sudden loss of health, resulting in hospitalization and a transition into the patient role [3]. This new role can cause emotional reactions such as anxiety by disrupting the patient’s homeostatic balance, due to the patient assigning meanings specific to themselves to the physical illness [4]. Anxiety is defined as the intense fear, worry and unease experienced by an individual in response to potential future dangers or threats. The condition is characterised by physiological changes, cognitive processes and behavioural responses, which can have a detrimental effect on an individual’s daily activities [5]. The emotional responses and changes that occur in intensive care patients are related to feelings of fear and anxiety associated with their physical illness, as well as stress factors such as loss of control, pain, delirium, sensory deprivation, social isolation, and fear of death. This situation can lead to anxiety [6].

Intensive care units are clinical units that provide round-the-clock care by specialised healthcare professionals to patients struggling with life-threatening health problems [7]. Such environments are characterised by an inherent level of stress, and for patients, the unfamiliar conditions and the presence of serious health problems and life-threatening situations can be a significant source of anxiety [8]. Individuals receiving intensive care are subject to numerous stressors, including invasive procedures, a diminution of privacy, separation from family members, immobility, pain, the necessity for mechanical ventilation, incessant noise, disorientation, frequent sleep interruptions, and the presence of strangers [9].

As has been documented, patients residing in intensive care units are susceptible to psychological distress, including anxiety [10,11]. Research conducted on the anxiety levels of patients in intensive care units has indicated that the prevalence of anxiety in the general patient group is 37.1% [12]. Conversely, a study encompassing patients receiving mechanical ventilation support revealed that the prevalence of anxiety escalated to 87.35%, signifying that ventilation support may exert a substantial influence on psychological distress [13]. A study indicated that nurses’ sensitivity to their patients’ emotional needs and open communication positively affected patients’ experiences and reduced anxiety [14]. A further study established that therapeutic approaches, such as music therapy, have the capacity to reduce anxiety levels in intensive care patients [15].

Nursing is a profession concerned with the utilisation of holistic approaches to improve health and overall well-being. Consequently, it is imperative for nurses to possess the ability to deliver comprehensive care, thereby facilitating the identification of patients’ anxiety levels and enhancing their psychological well-being [16]. In this regard, Aksoy et al. emphasised the importance of supportive environments in healthcare settings to reduce stress and anxiety in both patients and nurses [17]. Karadağ and Çiçek have observed that environments characterised by high levels of noise and constant interruptions can lead to an increase in feelings of anxiety among patients and hinder nurses’ ability to interact effectively with patients [18]. Consequently, in addition to acquiring skills through appropriate training and supportive environments, nurses must also hone their communication skills to effectively engage with patients. This ability is paramount in accurately assessing anxiety levels and alleviating these feelings. Effective communication is instrumental in accurately assessing patients’ anxiety levels and in alleviating these feelings through the provision of reassurance and support [19].

The approach of intensive care nurses to recognizing and addressing the anxiety experienced by hospitalized patients is critical to providing holistic care in intensive care units. Emotional distress is prevalent among critically ill patients, and nurses have a substantial role in addressing these psychological needs. This study aimed to explore intensive care nurses’ views and experiences regarding the recognition, assessment, and management of anxiety among hospitalized intensive care patients.

Accordingly, the study sought to answer the following research question: “What are intensive care nurses’ views and experiences regarding the recognition, assessment, and management of anxiety among hospitalized intensive care patients?”

## 2. Materials and Methods

### 2.1. Study Design, Setting, and Participants

The present study employed a qualitative descriptive design to explore intensive care nurses’ views and experiences regarding the recognition and management of anxiety in hospitalized patients. A qualitative descriptive approach was selected because it enables a comprehensive understanding of participants’ perspectives and clinical experiences within their natural context [20]. The Consolidated Criteria for Reporting Qualitative Research (COREQ) were followed in reporting the study [21].

The study was informed by a naturalistic, constructivist epistemological orientation. From this perspective, participants’ accounts were understood as context-dependent interpretations shaped by their clinical experiences, professional backgrounds, interactions with patients, and the organizational conditions of intensive care. Knowledge was therefore generated through the interaction between the participants’ accounts and the researchers’ interpretive analysis. During the interpretation of the findings, attention was given to the interrelated physiological, psychological, social, and environmental dimensions of intensive care nursing practice. This interpretive perspective supported a broader understanding of how nurses recognized and managed patients’ anxiety.

The study was conducted between July and September 2021 at a state hospital in the Marmara Region of Türkiye. Data collection took place during the COVID-19 pandemic, when intensive care practices were affected by pandemic-related conditions, including increased workloads, infection-control measures, restrictions on family visits, patient isolation, the use of personal protective equipment, and limitations in face-to-face communication. Therefore, the participants’ views and experiences should be interpreted within this specific clinical and historical context.

Of the 50 nurses working in the intensive care unit at the time of the study, those who were available during the data collection period and willing to participate were recruited using convenience sampling. Potential participants were approached sequentially and informed about the purpose and procedures of the study. None of the nurses who were approached declined participation or withdrew from the study. Data collection and preliminary analysis were conducted concurrently, and interviews continued until successive interviews produced no new codes, themes, or relevant insights. Data saturation was reached after 21 interviews; therefore, recruitment was concluded, and the remaining nurses were not approached. The sociodemographic characteristics of the 21 participants are presented in Table 1.

The purpose of the study was explained to the participants, and their voluntary written and verbal informed consent was obtained before data collection. Data were collected through face-to-face, individual in-depth interviews using a semi-structured interview guide. Participants were assigned anonymous codes to protect their identities, and the raw data were stored on an encrypted external drive.

The interviews were conducted and transcribed verbatim in Turkish, which was the shared language of the interviewer and participants. The quotations selected for inclusion in the English manuscript were translated into English by the research team during manuscript preparation. Although no formal back-translation procedure was conducted, the translated quotations were compared with the original Turkish transcripts to preserve their intended meaning and contextual accuracy.

Some participants entered nursing practice after graduating from a vocational health high school and subsequently completed an associate’s or bachelor’s degree while employed. Therefore, years of professional experience were calculated from their initial entry into nursing practice rather than from the completion of their highest academic degree.

### 2.2. Data Collection Tools

In the study, a semi-structured interview form was used for individual in-depth interviews to understand the participants’ experiences and expectations, along with an information form developed by the researcher to determine the characteristics of the individuals. At the beginning of the semi-structured interview form, participants were asked warm-up questions such as their age, marital status, educational status, years of work, and length of service in intensive care. The semi-structured interview guide consisted of 11 open-ended questions developed by the researchers based on a review of the relevant literature and expert opinions. Example questions included: “What do you think should be considered when a patient is first admitted to the intensive care unit?” and “What do you think about the impact of assessing intensive care patients’ anxiety levels on nursing activities?” The full interview guide is provided as Supplementary File S1. The interview guide was not formally piloted before data collection.

All interviews were conducted by E.T., a master’s-prepared researcher with four years of clinical field experience. E.T. had received training in qualitative research methods but had no prior experience in conducting qualitative interviews. At the time of data collection, E.T. was employed at the study hospital and was acquainted with the participants. However, she had no direct working, managerial, supervisory, or evaluative relationship with them. To minimize the potential influence of this prior familiarity and institutional affiliation, participants were informed that participation was voluntary, that they could withdraw without consequences, and that their responses would remain confidential and would not be shared with hospital administrators. Interviews were conducted privately, and identifying information was removed from the transcripts.

During the research process, data were collected through individual interviews using a semi-structured interview guide. The interviews were conducted at times convenient for the participating nurses and in locations of their choice. A total of 21 intensive care nurses were interviewed. Data collection and preliminary analysis were conducted concurrently, and the interviews continued until data saturation was reached. Data saturation was defined as the point at which successive interviews yielded no new codes, themes, or relevant insights. With the participants’ consent, all interviews were audio-recorded. Each interview lasted between 30 and 60 min, with a total interview duration of 880 min. The audio recordings were transcribed verbatim, resulting in 86 pages of textual data.

The data were analyzed manually using an inductive thematic analysis approach as described by Nowell et al. [22]. First, the interview transcripts were read multiple times to achieve familiarity with and a comprehensive understanding of the data. Meaningful data units were then identified, and initial codes were generated from the participants’ words and accounts. Two researchers independently reviewed the transcripts and developed preliminary codes. Their coding decisions were subsequently compared, and differences in coding or interpretation were discussed by returning to the relevant transcript excerpts. Agreement was reached through discussion and consensus.

Subsequently, related codes were grouped according to their similarities and differences and organized into categories. Themes and sub-themes were then developed to represent patterns within the data. The themes and sub-themes were reviewed, refined, and compared iteratively with the original data to ensure consistency, coherence, and accurate representation of the participants’ accounts. Representative participant quotations were used to demonstrate the relationship between the original data and the reported findings.

### 2.3. Data Validity and Reliability

The trustworthiness of the study was established according to Lincoln and Guba’s criteria of credibility, transferability, dependability, and confirmability [23]. These criteria provide a methodological framework for evaluating rigor and the quality of findings in qualitative research [24]. Credibility was supported through in-depth individual interviews, repeated reading of the transcripts, discussion of the codes and themes among the researchers, and the use of direct participant quotations to demonstrate the relationship between the findings and the participants’ accounts. Formal member checking was not conducted.

Transferability was supported by providing detailed descriptions of the participants, study setting, research context, and data collection procedures, thereby enabling readers to assess the applicability of the findings to other settings [25]. Dependability was enhanced by using the same semi-structured interview guide and following a consistent data collection procedure across participants. Two researchers independently reviewed the transcripts and generated preliminary codes. Their coding decisions were subsequently compared, and differences in coding or interpretation were resolved through discussion, return to the relevant transcript excerpts, and consensus. Confirmability was supported by retaining the semi-structured interview guide, audio recordings, anonymized transcripts, coding records, and theme-development documents. These materials allowed the researchers to trace the reported findings back to the original data.

## 3. Results

The study identified four main themes and ten sub-themes concerning intensive care nurses’ views and experiences regarding the recognition and management of anxiety in hospitalized patients. The main themes were “Nurses’ Initial Assessment Priorities,” “Nurses’ Recognition of and Responses to Patient Anxiety,” “Approaches to Reducing Anxiety,” and “Factors Affecting Anxiety Assessment.” The ten sub-themes were “Prioritizing the Emergency,” “Getting to Know the Patient,” “Under-recognition of Anxiety,” “Compliance with Care and Treatment,” “Recognizing Psychological Needs,” “Providing Information,” “Therapeutic Communication and Emotional Support,” “Workload,” “Organizational and Work-Related Factors,” and “Training Needs.” The themes and sub-themes are presented in Table 2.

### 3.1. Main Theme 1: Nurses’ Initial Assessment Priorities

All 21 participants contributed to the theme “Nurses’ Initial Assessment Priorities” while describing their experiences of recognizing and managing anxiety among intensive care patients. Within this theme, 17 participants referred to “Prioritizing the Emergency,” while 14 participants discussed “Getting to Know the Patient.”

#### 3.1.1. Sub-Theme: Prioritizing the Emergency

Under the sub-theme “Prioritizing the Emergency,” 17 of the 21 participants reported initially assessing patients’ vital signs, respiratory and circulatory status, and other urgent clinical conditions. They explained that although anxiety may be evident at admission, life-threatening and urgent clinical problems are addressed first, with attention shifting to the patient’s psychological state after clinical stabilization.

*“……… It is necessary to look at the overall picture to see if there is a risk to life. Vital signs need to be checked. The patient needs to be monitored as soon as possible. The level of consciousness needs to be assessed and the airway needs to be checked. For me, these are the urgent situations in the first stage.”*

(K19, Female)

*“First, attention should be paid to the urgent situation. For example, their vital signs and whether they are intubated are checked.”*

(K11, Female)

#### 3.1.2. Subtheme: Getting to Know the Patient

Under the sub-theme “Getting to Know the Patient,” 14 of the 21 participants reported assessing patients’ anxiety by observing physical and behavioral responses, including facial expressions, gestures, restlessness, sleep patterns, crying, and sweating. They emphasized considering patients’ psychological characteristics in addition to their physical symptoms. Representative participant statements included:

*“For those who have never been in intensive care before, it is a black box. When a person arrives, if they are conscious, their first thought is, ‘Am I going to die?’. In this situation, nurses should empathize and approach the patient accordingly. The patient should be introduced to the area, the nurse should introduce themselves, and the procedure should be explained. Once the moment of panic and curiosity passes, the patient will feel better. This situation also positively affects the response to treatment.”*

(K20, Female)

*“We must not forget that the patient has come to a new environment. First, I look at what is necessary for their life, I assess their breathing, I monitor them. Then I look at their state of consciousness. After the patient’s physical condition has stabilized, I do a psychological assessment. Here, I look at whether the patient is afraid or anxious and how they express this to us.”*

(K18, Female)

### 3.2. Main Theme 2: Nurses’ Recognition of and Responses to Patient Anxiety

The nurses participating in the study perceived that intensive care patients experienced anxiety and other psychological difficulties associated with environmental and clinical conditions, including continuous lighting, lack of windows, uncertainty, fear of death, equipment noise, invasive procedures, and loneliness. Participants’ accounts within this theme were organized into three sub-themes: “Under-recognition of Anxiety,” “Compliance with Care and Treatment,” and “Recognizing Psychological Needs.”

#### 3.2.1. Sub-Theme: Under-Recognition of Anxiety

Under the sub-theme “Under-recognition of Anxiety,” 12 of the 21 participants reported that monitoring vital signs, performing critical medical interventions, and addressing emergency clinical needs were their primary responsibilities. They explained that, within this context, patients’ emotional needs—including anxiety—were not always systematically assessed or prioritized. Participants also described heavy workloads and time constraints as factors that limited their ability to evaluate patients’ psychological states, resulting in anxiety sometimes being overlooked in clinical practice.

*“In general, patients are badly affected in intensive care. Patients are badly affected visually and audibly. We cannot assess them in great detail because our workload is too heavy and we have priorities.”*

(K16, Male)

*“It’s actually a procedure that absolutely needs to be done. While working during the pandemic, we saw the condition of many anxious patients deteriorate. When we look at it, we don’t really care about the patient’s mental state; we always try to improve their physical health. The system and working conditions force us to do this. But if the anxious patient isn’t improving and our goal is to heal the patient, then we need to evaluate it.”*

(K18, Female)

#### 3.2.2. Subtheme: Compliance with Care and Treatment

Under the sub-theme “Compliance with Care and Treatment,” 15 of the 21 participants reported frequently caring for patients whom they perceived as experiencing high levels of anxiety. According to the nurses, these patients sometimes exhibited resistance to treatment, difficulty cooperating with healthcare professionals, restlessness, and reluctance to follow recommended care and treatment procedures. Representative participant statements included:

*“……… If I calm the patient down, they don’t react to me and become more compliant. But if the patient’s anxiety level is high, they don’t allow us to perform certain procedures and sometimes become aggressive.”*

(K15, Female)

*“The patient must be assessed holistically during nursing activities. Therefore, whether or not they are anxious also affects our work. If the patient is anxious, we may not be able to provide treatment and care as we wish.”*

(K3, Female)

#### 3.2.3. Subtheme: Recognizing Psychological Needs

Under the sub-theme “Recognizing Psychological Needs,” 18 of the 21 participants emphasized the importance of assessing intensive care patients holistically and addressing both their physical and psychological needs. The nurses identified loneliness, fear of death, uncertainty, and feelings of dependence as important psychological experiences among intensive care patients. They perceived that recognizing and addressing these needs could support patients’ emotional well-being and facilitate their participation in care and treatment. Representative participant statements included:

*“Nurses are the primary professionals responsible for patient care. Therefore, they should provide appropriate care by assessing anxiety. Nurses implement the nursing process by diagnosing anxiety. In this way, they provide holistic care by assessing the patient from a biopsychosocial perspective.”*

(K21, Male)

*“Actually, as I mentioned in the previous question, if our goal is to heal the patient and restore their health, anxiety has a huge impact. Because health is a state of physical and mental well- being.”*

(K18, Female)

### 3.3. Main Theme 3: Approaches to Reducing Anxiety

The intensive care nurses emphasized that informing patients about the procedures to be performed and explaining the care processes could help reduce anxiety arising from uncertainty. Nurses’ approaches to reducing patients’ anxiety were categorized under the sub-themes of providing information and therapeutic communication and emotional support.

#### 3.3.1. Sub-Theme: Providing Information

Under the sub-theme “Providing Information,” 11 of the 21 participants reported that providing clear and explanatory information could help patients feel safer and experience a greater sense of control during procedures and the overall care process. The nurses emphasized that information should not be limited to medical procedures but should also address patients’ daily needs, expectations regarding care, and relevant aspects of the intensive care environment. Representative participant statements included:

*“Patients should be informed about the environment and what they may encounter before being admitted to intensive care. Because after admission, patients find it difficult to comprehend most of what is explained due to the tension created by the environment.”*

(K5, Female)

*“The patient should be given constant explanations. If they are conscious, permission should be obtained for every procedure we perform. There should be an environment where the patient can express themselves comfortably. The patient should not be afraid of the nurse, and we should make them feel that we understand what they are saying.”*

(K18, Female)

#### 3.3.2. Sub-Theme: Therapeutic Communication and Emotional Support

Under the sub-theme “Therapeutic Communication and Emotional Support,” 14 of the 21 participants emphasized the importance of listening attentively and attempting to understand patients’ feelings. They perceived that continuous and open communication could support patients’ psychological well-being and contribute to the quality of nursing care. Participants also described verbal and nonverbal interactions, including speaking calmly, maintaining eye contact, and using gentle touch, as approaches that could help alleviate patients’ anxiety. Representative participant statements included:

*“Patients with high anxiety should be communicated with. If the nurses in the field are insufficient, a meeting between the patient and the consultation-liaison psychiatric (CLP) nurse can be arranged. In addition to communication, breathing exercises can be taught and performed, music can be played, or communication with relatives can be facilitated to help them relax.”*

(K20, Female)

*“I think empathy should come first. Explaining things to the patient is very important. Because the patient is scared and becomes more stressed when they don’t know what is being done. Communication is very important here…”*

(K15, Female)

### 3.4. Main Theme 4: Factors Affecting Anxiety Assessment

The intensive care nurses reported that environmental stressors within the intensive care unit, including noise from medical equipment, confined spaces, frequent interventions, high mortality rates, and limited contact with family members, could increase patients’ anxiety. Factors influencing nurses’ ability to assess patients’ anxiety were categorized under the sub-themes of workload, organizational and work-related factors, and training needs.

#### 3.4.1. Sub-Theme: Workload

Under the sub-theme “Workload,” 14 of the 21 participants identified excessive workload as a barrier to assessing and managing patients’ anxiety. Within this group, 8 participants explained that competing clinical demands required them to prioritize urgent physical care, which could result in anxiety assessment and psychosocial care receiving less attention. Representative participant statements included:

*“We generally skip the anxiety part. Because that’s become our routine. Our priorities are different. We have a daily and intense shift schedule. In this situation, our priorities change.”*

(K6, Female)

*“Every other day shifts, our exhaustion, the more intensely we work, the less we communicate with patients. Because we are already tired.”*

(K10, Female)

#### 3.4.2. Subtheme: Organizational and Work-Related Factors

Under the sub-theme “Organizational and Work-Related Factors,” 12 of the 21 participants reported that the physical layout of the intensive care unit, confined spaces, equipment noise, crowding, and frequent clinical interventions could contribute to patients’ anxiety and make it more difficult for nurses to recognize and assess anxiety accurately. Representative participant statements included:

*“Factors such as individuals’ working hours, bullying, dissatisfaction with salary, and dislike of the job affect the individual’s approach to the patient, and the nurse’s personal problems are also very important”*

(K21, Male)

*“The workload, intense pace of work, number of patients per nurse, perhaps our psychology. Our psychology is actually affected by being confined to a closed space... Experience is also important. Communication with the patient is easier. Because you can predict what the patient might do next.”*

(K19, Female)

#### 3.4.3. Subtheme: Training Need

Under the sub-theme “Training Needs,” 14 of the 21 participants emphasized the need for additional in-service training on anxiety assessment and management. They believed that such training could strengthen their ability to respond to critical situations and facilitate the timely recognition and assessment of patients’ anxiety. Representative participant statements included:

*“Nurses should receive training on continuous communication. Working conditions should be improved. Nurses should be made to feel human, and team spirit should be established.”*

(K18, Female)

*“Nurses can be trained on this issue, or the institution can explain how nurses should proceed in such situations. Support can be sought from experts in the field.”*

(K20, Female)

Taken together, the four themes indicate that nurses’ recognition and management of patient anxiety in the intensive care unit followed a hierarchical and context-dependent process. Immediate physiological stabilization was prioritized during the initial assessment, while anxiety was generally considered after urgent clinical needs had been addressed. Nurses recognized anxiety through patients’ behavioural and emotional responses and attempted to reduce it by providing information, establishing therapeutic communication, and offering emotional support. However, excessive workload, environmental conditions, organizational constraints, and insufficient training limited the systematic assessment and management of anxiety. Thus, anxiety care appeared to depend largely on individual nurses’ awareness and professional experience rather than on a standardized assessment process.

## 4. Discussion

The present study explored intensive care nurses’ views and experiences regarding the recognition and management of anxiety among hospitalized patients. One of the most important findings was that nurses initially prioritized emergency clinical conditions and physiological stabilization, with attention shifting to patients’ psychological needs only after their immediate condition had stabilized. This prioritization is understandable in intensive care practice, where life-threatening problems require immediate intervention. Previous studies have similarly emphasized the importance of evaluating critical conditions at admission [26], conducting an initial patient assessment [27], and addressing psychological needs following physiological stabilization [28,29]. However, the findings of the present study extend this literature by indicating that anxiety assessment may remain secondary even after immediate stabilization rather than being systematically incorporated into the subsequent care process.

This finding highlights a tension between the biomedical priorities of intensive care and the provision of holistic nursing care. Although the participants were aware that critically ill patients experienced anxiety, its recognition appeared to depended largely on nurses’ observational skills, professional experience, and interpretation of patients’ physical and behavioral responses. Thus, the central issue was not simply that nurses appropriately prioritized emergency care at admission but that anxiety assessment could continue to be overlooked throughout the care process. This may be particularly important for patients who are unable to communicate clearly, show less visible distress, or present with anxiety-related symptoms that overlap with their critical illness. Although anxiety and depression are common among intensive care patients and their assessment is an important component of nursing care [30], awareness of anxiety may not necessarily result in its systematic evaluation or inclusion in an individualized care plan. Establishing rapport and becoming familiar with the patient may help nurses recognize subtle behavioral and emotional changes [31]; nevertheless, reliance on individual professional judgment alone may lead to variation in how anxiety is identified and addressed.

In the present study, nurses reported that patients in intensive care may experience anxiety due to environmental and physical factors such as constant lighting, social isolation, and device noise. These findings were reflected in the sub-themes of under-recognition of anxiety, compliance with care and treatment, and recognizing psychological needs. Consistent with the literature, alarm sounds, device noise, and conversations negatively affect patients’ psychological well-being [18,32]. In addition, factors such as inability to see loved ones, concerns about disease progression, fear of disability, and thoughts of death contribute to increased anxiety [33]. Previous studies also suggest that structured nursing interventions and emotional support can reduce anxiety and delirium in intensive care patients [10,11,34,35]. Overall, the findings indicate that environmental stressors play an important role in shaping patients’ psychological state in intensive care settings.

The literature indicates that communication in intensive care settings is often limited, leading to uncertainty, loss of identity, and communication difficulties among patients [36,37]. Consistent with this, nurses in our study emphasized the importance of therapeutic communication, emotional support, and providing clear information to reduce anxiety. While some participants mentioned supportive care practices, these were not consistently highlighted. Previous studies report that adequate patient information is associated with improved treatment compliance and reduced anxiety [14,19]. Patients in intensive care also face challenges such as isolation, disrupted sleep, unfamiliar environments, separation from family, and lack of information [38]. In contrast, family-centered interventions have been shown to reduce anxiety [39,40]. Overall, the findings suggest that anxiety in intensive care is influenced by multiple factors, including environmental conditions, communication, and psychosocial support, and that effective communication plays a key role in alleviating anxiety.

Workload, organizational and work-related conditions, and insufficient training were identified as barriers to the systematic assessment of anxiety. Excessive workload, long working hours, role ambiguity, and caring for critically ill or dying patients are recognized occupational stressors for intensive care nurses [41,42]. The physical conditions of intensive care environments may also increase psychological strain [43]. Within this context, the participants’ tendency to prioritize urgent physical care should not be interpreted solely as an individual omission. Instead, it may reflect organizational conditions that limit the time and resources available for psychosocial assessment. Previous evidence suggests that nurses whose educational needs are addressed are better able to recognize anxiety [44] and support the management of both patient and professional anxiety [45], whereas inadequate training may restrict anxiety assessment and intervention skills [46]. However, training alone may be insufficient if heavy workloads, staffing limitations, and workflow priorities continue to prevent nurses from incorporating psychosocial assessment into routine care. The findings therefore point to the need for both educational interventions and organizational arrangements that make anxiety assessment feasible in daily intensive care practice.

The findings of the present study should also be interpreted within the context of the COVID-19 pandemic, as the data were collected between July and September 2021. During this period, increased workloads, infection-control measures, restrictions on family visits, patient isolation, and the use of personal protective equipment may have influenced both patients’ experiences of anxiety and nurses’ ability to recognize and manage it. The participants’ direct references to working during the pandemic further support the relevance of this context. In particular, the findings concerning workload, separation from family members, limited communication, and the isolating intensive care environment may have been shaped or intensified by pandemic-related conditions. Nevertheless, these challenges are not exclusive to the pandemic and may also occur in routine intensive care practice. Therefore, the pandemic may have amplified existing environmental, organizational, and psychosocial barriers rather than being their sole cause. This context should be considered when interpreting the findings and assessing their transferability to non-pandemic intensive care settings.

### Limitations of the Study

This study has several limitations. First, it was conducted in a single intensive care unit, which may limit the transferability of the findings to other intensive care and clinical settings. The sample was also relatively homogeneous, as 19 of the 21 participants were women and 18 held bachelor’s degrees. Therefore, the findings may not fully represent the experiences of intensive care nurses with different gender, educational, institutional, and professional backgrounds. Variations in participants’ levels of intensive care experience may also have influenced their perspectives on anxiety assessment and management. Furthermore, because the data were based on self-reported experiences, the findings may reflect subjective interpretations shaped by the participants’ personal, cultural, and professional backgrounds.

Data collection continued until successive interviews produced no new codes, themes, or relevant insights. However, no formal saturation grid or separate documentary evidence of saturation was maintained. In addition, formal member checking was not conducted; therefore, participants did not verify the researchers’ interpretations of the data. Although two researchers independently reviewed the transcripts and resolved differences through discussion and consensus, the absence of participant validation may have limited the credibility of the findings.

The interviewer was employed at the hospital where the study was conducted and was acquainted with the participants, although she had no direct working, managerial, supervisory, or evaluative relationship with them. Despite the measures taken to protect voluntariness and confidentiality, this institutional affiliation and prior familiarity may have influenced participants’ willingness to disclose critical views or socially undesirable practices.

The data were collected during the COVID-19 pandemic, when intensive care environments were affected by increased workloads, infection-control measures, restrictions on family visits, patient isolation, and communication difficulties. These circumstances may have shaped or intensified the participants’ experiences; therefore, caution is warranted when transferring the findings directly to non-pandemic intensive care settings. Finally, approximately five years elapsed between data collection and the reporting of the findings. Intensive care practices, institutional conditions, visitation policies, and approaches to psychological care may have changed during this period. As no follow-up interviews were conducted, the current relevance and transferability of the participants’ views should be interpreted with appropriate caution.

## 5. Conclusions

The findings indicate that intensive care nurses recognize that patients experience anxiety; however, their recognition and management of anxiety are largely guided by individual professional experience rather than by systematic assessment. Participants reported that their initial priority during patient admission was addressing urgent clinical needs and that anxiety was generally assessed after the patient’s clinical condition had stabilized.

The nurses identified continuous lighting, equipment noise, the physical conditions of the isolation environment, separation from family members, limited communication, and insufficient information about the treatment process as factors that could increase patients’ anxiety. They also emphasized that effective communication and the provision of clear and explanatory information could help alleviate anxiety. Furthermore, workload, organizational and work-related conditions, and insufficient training in psychosocial care were identified as barriers to the systematic assessment of patients’ anxiety and its integration into individualized care plans.

In-service training programs addressing psychosocial care and the assessment and management of anxiety should be provided for intensive care nurses. Undergraduate and postgraduate nursing curricula should also place greater emphasis on psychosocial care and patient anxiety in intensive care settings. Healthcare institutions should support the use of standardized anxiety assessment tools and create appropriate organizational conditions for integrating psychosocial assessment into routine intensive care nursing practice.

## Figures and Tables

**Table 1:** Characteristics of Participants.

Participant	Gender	Age	Marital Status	Education Level	Years of Experience	Years of Experience in Intensive Care
K1	Female	24	Single	Bachelor’s	1	1
K2	Female	27	Married	Associate’s degree	9	1.5
K3	Female	24	Married	Bachelor’s	5	1
K4	Female	25	Single	Bachelor’s	1.5	1.5
K5	Female	24	Single	Bachelor’s	1.5	8 months
K6	Female	37	Married	Bachelor’s	12	8
K7	Female	26	Married	Bachelor’s	2.5	1.5
K8	Female	28	Single	Associate’s degree	9	5
K9	Female	22	Single	Bachelor’s	2	1.5
K10	Female	38	Married	Bachelor’s	15	5
K11	Female	25	Married	Bachelor’s	3	3
K12	Female	25	Single	Bachelor’s	4	6 months
K13	Female	25	Single	Bachelor’s	2	15 months
K14	Female	22	Single	Bachelor’s	1.5	1.5
K15	Female	32	Married	Associate’s degree	14	2.5
K16	Male	23	Single	Bachelor’s	10 months	10 months
K17	Female	25	Single	Bachelor’s	1	1
K18	Female	24	Single	Bachelor’s	19 months	1
K19	Female	40	Married	Bachelor’s	18	12
K20	Female	26	Single	Bachelor’s	3	1.5
K21	Male	23	Single	Bachelor’s	2	2

**Table 2:** Themes emerging from the interviews.

Theme	Subtheme
NURSES’ INITIAL ASSESSMENT PRIORITIES	Prioritizing the Emergency Getting to Know the Patient
NURSES’ RECOGNITION OF AND RESPONSES TO PATIENT ANXIETY	Under-recognition of Anxiety Compliance with Care and Treatment Recognizing Psychological Needs
APPROACHES TO REDUCING ANXIETY	Providing Information Therapeutic communication and emotional support
FACTORS AFFECTING ANXIETY ASSESSMENT	Workload Organizational and work-related factors Training Needs

## Data Availability

The data presented in this study are available upon request from the corresponding author due to participants’ privacy and ethical requirements.

## Supplementary Materials

The following supporting information can be downloaded at: https://www.mdpi.com/article/10.3390/healthcare14193301/s1, Supplementary File S1: Interview Form.
