# Supplementary material for: Intensive Care Unit Nurses’ Views on Recognizing and Caring for Inpatients’ Anxiety Levels: A Qualitative Descriptive Study
Source: Healthcare (Basel). 2026 Oct 3;14(19):3301. doi: 10.3390/healthcare14193301 (PMC13665097; doi:10.3390/healthcare14193301)
Supplement: Supplementary file 1 [file healthcare-14-03301-s001.zip › healthcare-4589740-supplementary.pdf]

Supplementary File S1: Semi-Structured Interview Form

1-INTRODUCTION (INTRODUCTION - STATEMENT OF PURPOSE)

Greetings. I am Esin TEKİN, I am a postgraduate student in psychiatric nursing at Kocaeli University.

Our interview will take approximately 30-60 minutes. With your permission, I would like to record the interview. This way, I will not waste time writing down what you say. Because every word you say is very valuable, and I do not want to forget where you experience emotional intensity along with what you say. Everything you say during this interview will be used solely for scientific purposes, and your personal information will remain confidential when the research results are written. The information obtained as a result of this study may only be used for academic publication purposes. The information you provide will be evaluated together with the information provided by other participants, and your name will not appear anywhere. Is this acceptable to you? If you do not give your consent, I will take notes of what you say and will not record the interview.

Participation in the research is entirely voluntary. If you do not wish to continue with the research, you may leave at any time. Reading and approving this form, or answering the questions, will mean that you agree to participate in the research. Thank you for taking part in this research. If you have any questions before we begin the interview, I will be happy to answer them. If you give your permission, I would like to begin the interview.

1. What factors must be taken into consideration during the initial admission of a patient to the intensive care unit?

2. What are your thoughts on the assessment of anxiety in ICU patients?

> **2.1.** What is your opinion on the impact of evaluating patients in the intensive care unit for anxiety levels on nursing activities?

3. What are your thoughts on how to approach patients in terms of anxiety?

> **3.1.** It is requested that the positive and negative feedback that would be observed in patients following the implementation of these interventions be outlined.

4. What factors do you consider to be influential in the implementation of anxiety management approaches by intensive care nurses?

5. What measures should be implemented to enhance the recognition and management of anxiety in patients?

6. How do you determine whether the interventions you implement are effective? What positive or negative changes do you observe in patients?

7. What challenges do you encounter in the process of assessing and managing patient anxiety?

8. How do the physical conditions of the intensive care unit, workload, teamwork, or institutional practices affect anxiety management?

9. How do you think the presence or absence of patients' relatives affects the anxiety management process?

10. Do you feel competent in assessing and managing anxiety? In which areas do you think you need training or support?

11. What would you recommend doing to ensure more effective assessment and management of patient anxiety in intensive care units?

Probing Questions

- Can you give an example of this situation?
- What does this mean to you?
- What was the reason for feeling that way?
- What approach did you take in that situation?
- How did the patient react?
- Did you do anything differently?
- What was the impact of this on the care process?
- Is there any other experience or opinion you would like to share?
